# Advanced Pharmaceutical Applications of Hot-Melt Extrusion Coupled with Fused Deposition Modelling (FDM) 3D Printing for Personalised Drug Delivery

**DOI:** 10.3390/pharmaceutics10040203

**Published:** 2018-10-24

**Authors:** Deck Khong Tan, Mohammed Maniruzzaman, Ali Nokhodchi

**Affiliations:** Pharmaceutics Research Laboratory, Arundel Building, School of Life Sciences, University of Sussex, Brighton BN1 9QJ, UK; D.tan@sussex.ac.uk (D.K.T.); m.maniruzzaman@sussex.ac.uk (M.M.)

**Keywords:** hot-melt extrusion, 3D printing, drug delivery, personalised medicine, bioavailability

## Abstract

Three-dimensional printing, also known as additive manufacturing, is a fabrication process whereby a 3D object is created layer-by-layer by depositing a feedstock material such as thermoplastic polymer. The 3D printing technology has been widely used for rapid prototyping and its interest as a fabrication method has grown significantly across many disciplines. The most common 3D printing technology is called the Fused Deposition Modelling (FDM) which utilises thermoplastic filaments as a starting material, then extrudes the material in sequential layers above its melting temperature to create a 3D object. These filaments can be fabricated using the Hot-Melt Extrusion (HME) technology. The advantage of using HME to manufacture polymer filaments for FDM printing is that a homogenous solid dispersion of two or more pharmaceutical excipients i.e., polymers can be made and a thermostable drug can even be introduced in the filament composition, which is otherwise impractical with any other techniques. By introducing HME techniques for 3D printing filament development can improve the bioavailability and solubility of drugs as well as sustain the drug release for a prolonged period of time. The latter is of particular interest when medical implants are considered via 3D printing. In recent years, there has been increasing interest in implementing a continuous manufacturing method on pharmaceutical products development and manufacture, in order to ensure high quality and efficacy with less batch-to-batch variations of the pharmaceutical products. The HME and FDM technology can be combined into one integrated continuous processing platform. This article reviews the working principle of Hot Melt Extrusion and Fused Deposition Modelling, and how these two technologies can be combined for the use of advanced pharmaceutical applications.

## 1. Introduction

The 3D printing technology was first introduced by Charles Hull in the early 1980s [1]. It is a manufacturing process that creates a three-dimensional object layer-by-layer from a 3D digital model that can be generated from computer-aided design (CAD) software. Although the technology was developed in the 1980s, 3D printers were not widely available commercially until the early 2010s [2]. During the early stages, 3D printing was primarily used for making prototypes of products due to its cost-effectiveness and short production time. It was mainly used in various disciplines for the design and testing phase in the development of a new product. Three-dimensional printing has major advantages over traditional rapid prototyping processes, as the materials used in 3D printers are less expensive and it is able to provide a faster mode of iteration [2]. The rapid growth and improvement in the 3D printing technology have made it not just a rapid prototyping technique, but also a preferred production method in major industries [3,4,5]. Therefore, 3D printing is considered a very cost-effective manufacturing process, especially in producing objects with high complexity. It is now a preferred method to produce custom-designed objects, which could be very expensive and time-consuming using conventional manufacturing techniques such as injection moulding, machining and casting [5,6,7,8]. Nowadays, 3D printing technology is being used across various industries including architecture [9], construction [10], automotive [11], food [12], fashion industry [13], medical sector [1] and many others. The 3D printing technology has gained much popularity in the healthcare and medical field not just for its cost-effectiveness but also for the manufacturing flexibility. It is very useful in creating devices or medicine that are tailor-made for specific patients. The healthcare market in using 3D printing is expected to show significant growth due to increasing demand for custom-tailored and patient-specific medical products. The ability of 3D printing to produce rapidly only with a CAD model, allows for on demand-fabrication, creating the possibility for medical devices to be produced in-house. Custom made prostheses, hearing aids and some implants have been successfully made using the 3D printing technology. It can also fabricate the 3D constructs from a patient’s own medical images, such as computed axial tomography (CAT) and magnetic resonance imaging (MRI) [14]. These parts could be very useful for surgeons to practice on before performing a surgery on the real patient, and can also be used as a guidance and explanation of the operation procedure to the patient [15].

The Hot-Melt Extrusion (HME) technology was established in the early 1930s and was originally used for the manufacturing of plastics and rubber products [16]. This technology can be used to produce the filaments that are required to be used in Fused Deposition Modelling (FDM) 3D printers. In recent years, this technology has gained much interest in the pharmaceutical industry, especially for the production of oral dosage forms and drug delivery systems. Apart from that, drug loaded filaments which are suitable for 3D printing has also been produced. In the HME process, active pharmaceutical ingredients (APIs) are blended with a thermoplastic polymer and then extruded as filaments that are used in 3D printing. This article will provide a review on the mechanism of HME, FDM 3D printing and integrating HME with FDM 3D printing as a continuous process to produce pharmaceutical dosage forms.

## 2. Mechanism of Hot-Melt Extrusion (HME)

**Figure 1:**
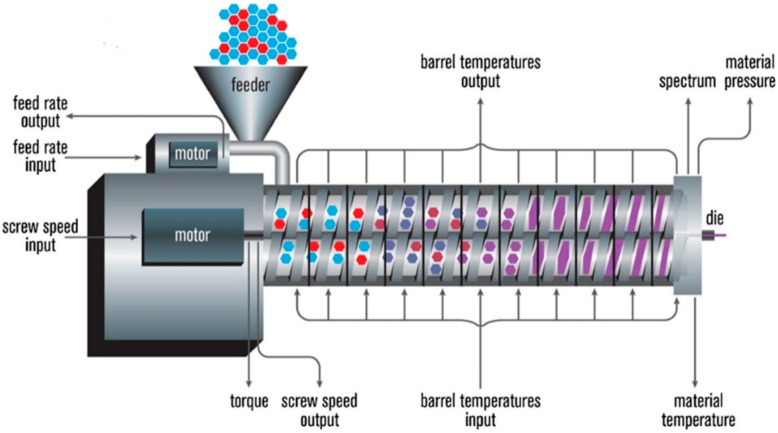
A schematic diagram of a typical hot melt extruder (Reproduced with permission from reference [17], Springer, 2015).

HME is a continuous process where heat and pressure are applied to melt or soften materials through an orifice to produce new products of uniform shape and density. The extrusion process can change the physical properties of a substance when it is being forced through an orifice or die on the hot-melt extruder under controlled conditions [17]. The main component of HME is the extruder. Some of the basic elements that are assembled to make an extruder include a motor, an extrusion barrel, rotating screws in the barrel and a die or orifice that is connected at the end of the extruder [17]. The extrusion barrel containing the rotating screws is often made up of several sections that are bolted together. The extruder contains heaters that provide heat for the melting or softening of the materials. The screws in the extruder can provide shear stress and intense mixing of the materials. The friction created by the screws in the barrel and the heat provided cause the polymeric material to melt. The screw then conveys the melted material down the barrel. The extruder is controlled through a central electrical control, which is connected directly to the extrusion unit. Some of the processing parameters that can be controlled are screw speed in revolutions per minute (RPM), feed rate, temperature along the barrel and the die, and the vacuum level for devolatilization [17]. A schematic diagram of a typical extruder is illustrated in Figure 1.

**Figure 2:**
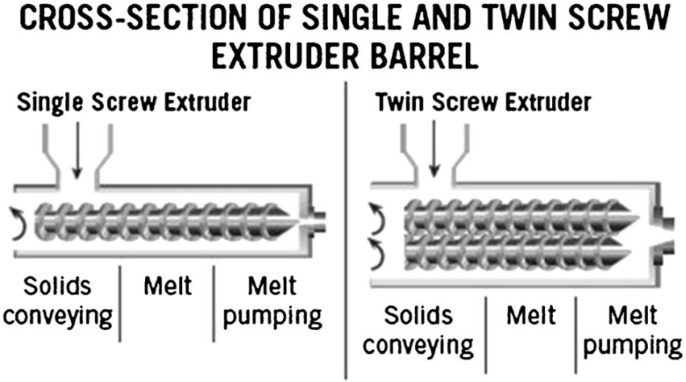
Cross-section of a single and twin-screw extruder (Reproduced with permission from reference [17], Springer, 2015).

In general, there are two types of hot-melt extruders: the single-screw extruder and twin-screw extruder (Figure 2). Some of the advantages and disadvantages of the single-screw extruder and the twin-screw extruder is compared in Table 1. The primary purpose of a single-screw extruder is to melt polymers and extrude them into a continuous shape. In the single screw extruder, there is only one screw in the extruder, which is used for feeding, melting, devolatilising and pumping [18]. Materials are fed into the extruder from the feeder/hopper and then transported down the barrel using the screw. Most single screw extruders are flood fed, which means the feeder sits over the feed throat and the speed of the screw determines the output rate. Occasionally, the single screw extruder can also be starve fed, where a feed system sets the mass flow rate and the output rate is independent of the screw speed [18]. When the extruder is starve fed, it is fed at a rate less than the forwarding efficiency of the screws [19]. When the screw rotates in the extruder barrel, frictional forces are created between the rotating screw and the surface of the barrel, forming a flow channel that is responsible for transporting the material fed down the barrel to the proximal end of the screw [17]. Increasing the screw speed can increase the frictional forces created, resulting in higher heat energy created. The shearing caused by the rotating screw and the heat energy produced from the heaters will melt the material, which will then result in a melt pool of the material. The molten material will then be pumped through a die, which will give a fixed shape that forms the extrudate [18]. However, high pressure may be created during the melting process in a single-screw extruder. The high pressure will compress the dispersed particulates in the molten material, which could cause agglomeration and poor mixing due to insufficient shear deformation.

**Figure 3:**
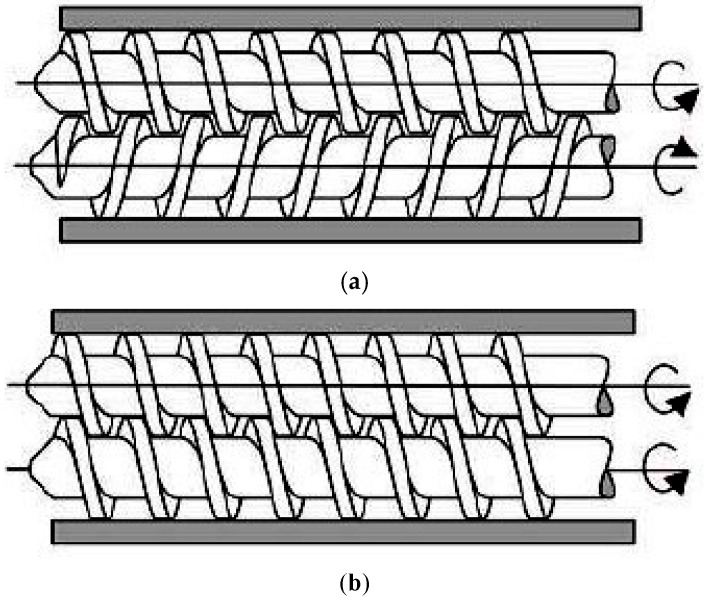
Twin-screw extruder barrel (**a**) Counter-rotating screws (**b**) Co-rotating screws (Reproduced with permission from reference [29], Scientific & Academic Publishing, 2016).

On the other hand, the twin-screw extruder uses two screws arranged side-by-side in the barrel. The twin-screw extruder is used mainly for the purpose of mixing two or more materials together to form a blend. The additional materials that could be melted and mixed with polymers are, for example, pigments (e.g., iron/chromium oxide [20]), fillers (e.g., lactose, starch [21]), APIs (e.g., acetaminophen [22]) and plasticisers (e.g., polyethylene glycols, Triacetin [21]) [23]. Most of the time, a twin-screw extruder will be starve fed with the raw materials. This means that the output rate of the extrudates will depend on the feeder and independent of the screw speed [24]. In a twin-screw extruder, the screws can either be co-rotating (screws rotate in the same direction) or counter-rotating (screws rotate in the opposite direction) (Figure 3). The counter-rotating screws can produce very high shear forces as the materials are squeezed into the gaps between the two screws [18]. These two types of twin-screw extrusions can further be classified into fully intermeshing or non-intermeshing. The fully intermeshing twin-screw extruder has a special self-cleaning feature, which can help reduce the non-motion during the extrusion process and also prevents localised overheating of the raw materials when inside the extruder. This is because the raw materials do not rotate along the screw in the intermeshing screws configuration. The flight of one of the screws will be able to clean the root of the adjacent screw, removing all the materials away from the extruder assembly during the extrusion process. This can also reduce any product waste that could happen at the end of a production batch. The non-intermeshing extruder has a weaker screw interaction and lower self-cleaning capability, making it not as popular as the intermeshing screws for material mixing applications. The non-intermeshing screws are often used to process highly viscous materials and can be used to remove large amounts of volatile substances. The non-intermeshing screws are positioned separately from each other, resulting in a lower torque generation, which is suitable for processing highly viscous materials [17].

For twin-screw extruders, the shearing of the materials occurs between the rotating screws and between the screws and the wall of the barrel. The torque and shear created by the rotating screws produce heat energy that is used to melt the materials. When the melted material is being squeezed between the gaps of the rotating screws, it is being continuously stretched as a rubber band, causing viscous heating dissipation to occur [24]. The heat generation in a twin-screw extruder is independent of the screw speed. The configurations of the twin screw can be adjusted depending on the desired level of shear and mixing. This means that there is a better control of the melting of the materials in a twin-screw extruder. Hence, there is a lower tendency of overheating, which is particularly useful when processing heat sensitive materials [17].

The main feature of a twin-screw extruder is the enhanced and homogenous mixing of materials during extrusion. During twin-screw extrusion, two types of mixing can occur (a) distributive mixing and (b) dispersive mixing. Distributing mixing involves melt division and recombination. This means that the materials can be evenly blended with minimal degradation. Apart from that, the size of the materials is reduced in distributive mixing. Distributive mixing allows for the use of heat and shear sensitive materials such as APIs. Dispersive mixing involves shear and elongational mixing [24]. It induces solubilisation of one component to another component. Dispersive mixing breaks down the droplet or solid materials to fine particles using high energy. The enhanced mixing feature in the twin-screw extruder contributes to the viscous heat dissipation. These make the twin-screw extruder more efficient in heating the materials, and hence, it can readily process thermoplastic polymers (e.g., hydroxypropyl cellulose) [24]. Some other advantages of a twin-screw extruder over a single-screw extruder are easier material feeding, high kneading and dispersing capabilities, lower tendency of overheating and shorter transit time [17]. As twin-screw extrusion provides uniform and homogenous mixing, the twin-screw extrusion process can be easily scaled up and optimised. The possibility of scaling up the extrusion process makes it a more efficient process for large volume production. Therefore, the majority of the food and plastics industries have been using twin-screw extrusion for their production [25,26,27]. Apart from that, twin-screw extrusion has also been adopted as a manufacturing process in the pharmaceutical field [28]. However, there might be an issue for pharmaceutical industry when it comes to the development of a formulation containing some expensive drug/material.

**Figure 4:**
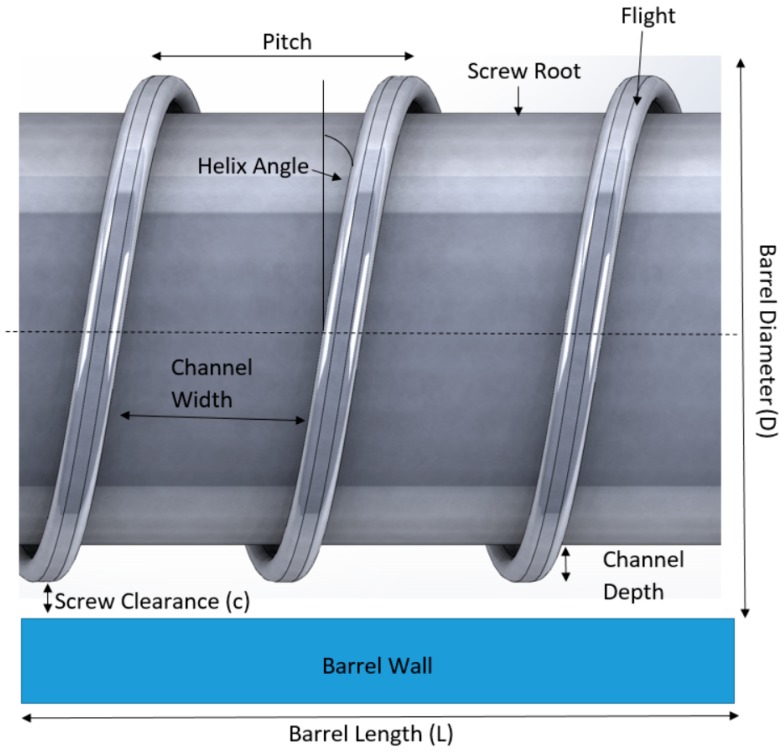
Parameters of a screw in a hot-melt extruder.

**Figure 5:**
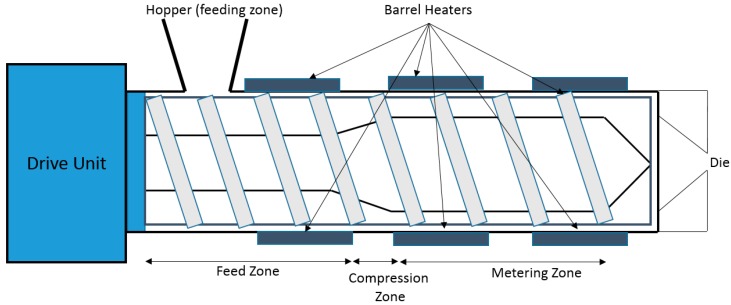
Three different zones of a screw in an extruder: Feeding, compression/melting and metering zone.

The screw in the hot-melt extrusion process is very important in determining the mixing and the extrusion process. Figure 4 shows the parameters of a typical screw used in a hot-melt extruder. The dimensions of the screws are normally defined in length-to-diameter (L/D) ratio. Most screws are coated with stainless steel which could reduce friction and avoid chemical reactions with the raw materials. The screw is typically divided into three sections along the length of the barrel: the feed section, melting/compression and metering (Figure 5). At the feed section, the screw transfers the materials from the hopper to the barrel. The screw in the feed section has flights that are deeper or of a greater pitch, and the channel depth is also wider. This allows for the raw material to fall easily into the screw for conveyance along the barrel and to facilitate mass flow. The pitch and helix angles determine the output of the extruder at a constant screw speed. The solid raw materials are then conveyed to the compression zone for mixing, compressing, melting and plasticising. The channel depth decreases the compression zone in order to increase the pressure in the barrel which could remove any entrapped air. The polymers will then soften and melt in the compression zone. As the material enters the metering zone, the material should be in molten state. The metering section is to reduce the pulsating flow and to ensure a uniform delivery rate through the die [17,18].

The last section of the extruder is the die or orifice. The function of the die is to shape the molten material when it is exiting the extruder. It is designed to achieve the required shape and dimensions of the extrudate. The cross-section of the extrudate will increase upon leaving the die and this phenomenon is known as “die swell” [18]. This phenomenon depends on the viscoelastic properties of the polymers [30]. In general, there can be four different shapes for products made by extrusion dies including the extrudate strands, films, sheets and granules [17].

## 3. Hot-Melt Extrusion in Pharmaceutical Applications

**Figure 6:**
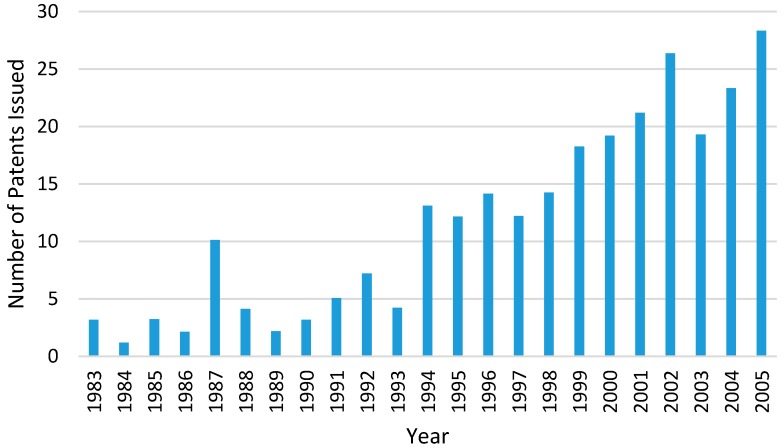
Number of Hot-Melt Extrusion patents issued for pharmaceutical applications between year 1983 to 2006 (the data for chart is extracted from reference [32], Taylor & Francis, 2008).

Hot-melt extrusion is a continuous manufacturing process that includes several operations such as feeding, heating, mixing and shaping into one continuous process. There has been growing interest in implementing continuous manufacturing processes on pharmaceutical product in the pharmaceutical industry [24]. Therefore, the HME technology has received much attention from the medical and pharmaceutical industry for the production of pharmaceutical dosage forms and medical implants [31]. The number of HME patents issued for pharmaceutical applications has increased significantly since the 1980s (Figure 6) [32]. This is because the HME process is able to meet the regulatory requirements in the manufacturing process of dosage forms and also have large flexibility, particularly for the increasing demand for personalised medicines. Regulatory bodies such as the U.S. Food and Drug Administration’s (FDA) encourages manufacturing processes that involves process analytical technology (PAT) in order to enhance product and process understanding. The initiative of PAT is to closely monitor some key parameters during the manufacturing process which could help to optimise design, analysis and control within the manufacturing process [19,30]. This is can be demonstrated and fulfilled in the HME process.

During the hot-melt extrusion process of pharmaceutical dosage forms, APIs, thermoplastic polymeric carrier and processing aids such as plasticisers and antioxidants, are heated and mixed in the extruder and then being forced through a die into shapes including granules, cylinder or films [33]. In recent years, much work has proven that HME has the ability to improve the solubility and bioavailability of poorly soluble drugs [34]. For instance, the bioavailabilty of poorly soluble APIs (e.g., Ritonavir, Troglitazone, Anacetrapib, Suvorexant) can be improved when being used a type of formulation called the solid dispersion which can be prepared using HME [16,34]. A solid dispersion is a system where one or more APIs are molecularly distributed into a hydrophilic inert carrier matrix. The matrix can either be crystalline or amorphous, and the APIs will be dispersed molecularly in crystalline particles or amorphous particles [17,35]. In the early 1990s and 2000s, the Rezulin^®^ and Kaletra^®^ tablets have been successfully produced using the HME process, which have shown improved bioavailability. These two tablets were produced using twin-screw extrusion as it can provide homogeneous and consistent mixing of multiple ingredients [24]. Since then, the HME process, and in particular, the twin-screw extrusion, has gained much popularity in the pharmaceutical industry.

The powerful processing technology of HME has also attracted much attention in developing different types of drug delivery systems, as it has opened up the opportunity to use some of the molecules that were previously seemed not suitable to be made into pharmaceutical dosage forms [32]. Its ability and efficiency in producing solid dispersions has also made it possible for the development of sustained, modified and targeted drug delivery systems. The different drug release systems can be achieved by controlling the formulations and the processing parameters when using HME [17].

As seen is some early studies, HME has been used to develop sustained release matrix mini-tablets manufactured from ethyl cellulose, HPMC and ibuprofen [36,37]. Vegetable calcium stearate (CaSt) was also seen reported in the development of sustained release pellets using as a thermoplastic excipients [38]. A microbicide intravaginal ring (IVRs) IVR was also prepared and developed from polyether urethane (PU) elastomers for the sustained delivery of UC781 [39]. There has been also a study where HME was used to produce sustained-release solid lipid matrices of diclofenac sodium (Df-Na), and the extruded products were being compressed into tablets [40]. A lipophilic excipient, Glyceryl dibehenate (Compritol 888 ATO (C-888)) was used for the development of Df-NA sustained-release lipid matices. The purpose of the study is to investigate the effects of some parameters (e.g., extrusion temperatures, formulation composition, drug-lipid ratio) on the drug release behavior. It is found that Df-Na can either be in the crystalline or amorphous state depending on the processing conditions. The dissolution rate is affected by the drug–lipid ratio and the extrusion temperatures. There was an excellent distribution of Df-Na on the surface of the compressed tablets. The study has proven that Df-Na sustained-release tablets can be effectively produced by processing solid lipids using HME [40]. 

Bruce et al. have successfully developed tablets to target deliver 5-aminosalicylic acid (5-ASA) to the colon [41]. The tablets, prepared using the HME technique, are able to homogeneously disperse the crystalline 5-ASA throughout the polymeric matrix. The polymeric carriers used are Methacrylic Acid—Methyl Methacrylate Copolymer (1:2) and Eudragit^®^ S 100. Two types of plasticisers (Triethyl Citrate (TEC) and citric acid) were used in the study. TEC was able to reduce the glass transition temperature of the polymer, thus lowering the processing temperature. It can also affect the drug release rates of the tablets due to the leaching of TEC during the dissolution testing. The citric acid causes a slower drug release rate as it causes a decrease of the pH in the microenvironment of the tablet, which suppresses the polymer ionisation. The drug release profiles of the tablets were found to fit both the diffusion and surface erosion model [41].

Apart from developing drug delivery systems with different release patterns, HME can also be used to create drugs that could mask the bitterness of APIs. There are still a significant number of drugs on the market that have very poor organoleptic properties, which makes it unpalatable, especially for paediatric and geriatric medications. In order to improve the palatability of the drugs, taste masking has been an important consideration for the development of a formulation [42]. There are works that have shown that pharmaceutical dosage forms produced using the HME process can mask the bitter taste of APIs. This can be done by mixing the taste-masking polymer with the APIs during the production of a solid dispersion. The solid dispersion can prevent the interaction between the drug molecules and the taste bud by preventing the release of bitter drugs in the saliva [17]. Eudragit E-PO has been widely used as a taste-masking excipient in formulations, as it is insoluble below pH 5, thus preventing the release of the drug in the mouth [42].

When an extruder is used for pharmaceutical applications, it is required to meet some regulatory requirements. Therefore, some parts are different from the extruder which is used for manufacturing plastics. The metallurgy of the contact parts must not be reactive, additive or absorptive with the product, and these parts are made of medical grade steel. The oils and lubricants used are also fully FDA-conformant. The equipment needs to be configured for the cleaning and validation requirements associated with a pharmaceutical environment [18]. A comprehensive documentation of the process is required for monitoring and controlling the parameters such as feed rate, temperature, screw speed, pressure, melt viscosity, drive amperage and torque. The temperature in the barrel needs to be optimised so that the viscosity of the melted material is low enough to be transported down the barrel, but still sufficient for proper mixing. Apart from that, the temperature should also be kept low enough to avoid thermal degradation of the materials. There are also good manufacturing practices (cGMP) guidelines for the cleaning of HME used for pharmaceutical applications. The extruder for pharmaceutical purposes have been redesigned to enhance the ease of disassemble and limit the dead spots, allowing for efficient cleaning and maintenance. The equipment must be cleaned at appropriate intervals and written procedures are required that must be specific and detailed. Cleaned equipment must be protected from contamination while in storage and inspected before being used. Records and equipment logs of all cleaning and inspection must be kept. The time between the end of processing and cleaning steps must be recorded [19]. During cleaning, the extruder assembly is disassembled and any materials present on the screw, barrel and die need to be removed. The surfaces then need to be swabbed and analysed to satisfy cleaning validation requirements [33].

There are many advantages in using HME for pharmaceutical applications, which makes the technology more popular as a manufacturing method for pharmaceutical products. HME can be modified to include appropriate PAT tools, making it a continuous manufacturing process, which is a fabrication method that is highly encouraged by FDA as it meets the goal of PAT scheme to improve the efficiency and effectiveness of manufacturing process design, control and quality assurance [43]. One other reason for HME to be a preferred method is that the process does not require any use of solvent when producing solid dispersions. Traditional formulation methods, such as roll spinning and spray drying, require the use of organic solvents. These organic solvents can cause environmental issues when being disposed. As HME is a solvent-free process, this makes it an environmentally friendly process, making it a preferred process over conventional methods [42]. Some other advantages of using HME in pharmaceutical applications include shorter production time, higher process efficiency, and increases drug delivery efficiency in patients [33]. The biggest advantage of using HME in pharmaceutical is the prospect of being able to create new and novel formulations through this technique.

However, there are a few drawbacks in the HME process [43]. HME requires high energy input, which is required for the shear forces and the elevated temperature in the barrel of the extruder. The high temperature may also cause thermal degradation of the APIs and the high shear forces may also cause mechanical degradation to the polymers. However, there has been ongoing research work to overcome these issues, and they can be solved by proper formulation and equipment design, as well as different engineering approaches [32].

## 4. Mechanism of Fused Deposition Modelling (FDM)

**Figure 7:**
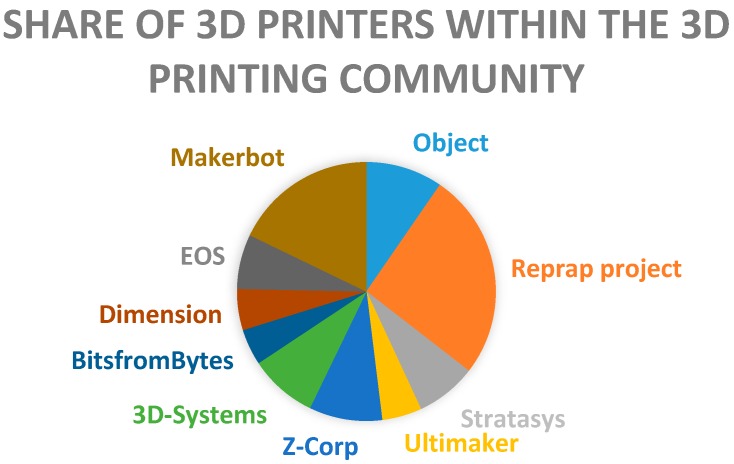
Share of most commonly used 3D printer manufacturers within the 3D printing community (data for chart extracted from reference [45], First Monday, 2013).

**Figure 8:**
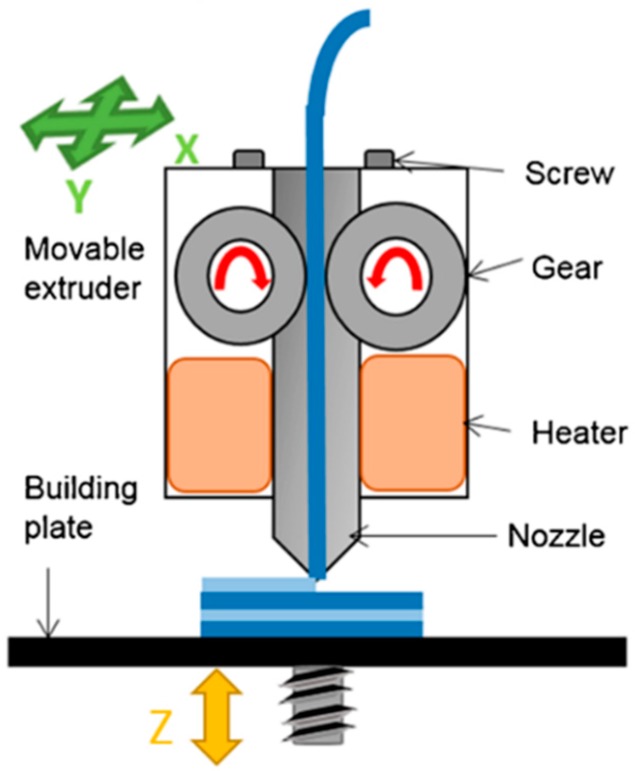
Mechanism of a Fused Deposition Modelling (FDM) 3D printer (Reproduced with permission from reference [47], Springer, 2016).

FDM is considered the most low cost and easily accessible 3D printing technology. FDM based 3D printers are so common that they are even found in homes for personal use, such as normal inkjet printers. FDM is one of the 3D printing techniques that is available in open source, which makes this technology the most affordable, as compared to others such as selective laser sintering (SLS), powder-based 3D printing, and stereolithography (SLA). This is because the open source 3D printing is shared freely with the public and can be easily built by anyone, making it possible for anyone to have an FDM 3D printer as a common household object. RepRap is a 3D printing community that started the open source 3D printer revolution [44]. A survey within the 3D printing community that was done in 2012 shows that RepRap is the most commonly used 3D printer manufacturer [45] (Figure 7). In FDM 3D printers, thermoplastic materials are typically used as the starting material. These thermoplastics materials that are fed into an FDM printer are usually in the form of a filament. The thermoplastic filaments are melted or softened, and then extruded from a printer head that is able to move in the x- and y-direction. The object is formed by many layers of melted thermoplastic. Each layer fuses and bonds to the layers below [46]. The layers of melted thermoplastic solidify almost immediately after being extruded from the print head. As one layer cools down and solidifies, another layer of the melted filament will be deposited on top of it until the printing of the object is finished (Figure 8).

Acrylonitrile Butadiene Styrene (ABS) and Polylactic Acid (PLA) are the two most common thermoplastics used in FDM machines [48]. High performance thermoplastics such as Polyether Ether Ketone (PEEK) and Polyether Imide (PEI) are some of the newer thermoplastics that are currently used in FDM printers [49]. These materials usually have very high melting temperature and excellent mechanical properties. They have been considered to be a cost-effective alternative to metal in some applications, as they are strong and also light [50]. PEI is a durable thermoplastic that exhibits physical properties such as high heat, solvent and flame resistance, high dielectric strength, thermal conductivity and overall strength. It has a lower impact strength and working temperature than PEEK [51]. Some of the PEI materials that are compatible with 3D printing have been used to produce plastic parts for aircraft [51]. Table 2 shows the comparison of different types of thermoplastics that are currently being used in FDM 3D printing. There are many challenges in using high-performance thermoplastics for FDM 3D printing due to their chemical and physical properties. As these materials possess very high melting temperature, the printing environment needs to be in a high temperature as well. This means that not just the extruder needs to be kept at the melting point of the material, but that the printing chamber needs to be kept at an evenly distributed high temperature as well during the printing process. This is because any cool areas will cause the material to shrink unevenly, resulting in warping or even a lack of bonding in between the layers of the materials [51]. One of the 3D printers that has been successfully developed by a 3D printing company called INTAMSYS is the FUNMAT HT (Shanghai, China) is capable of printing various 3D printing materials including ABS, PLA, PEI, etc. [52].

Some post-processing might be needed for a 3D printed object using the FDM method as the surface finish of the object is often quite rough due to the layering of the melted thermoplastics. Acetone is often used to smoothen ABS so that it has a glossy finish. The 3D printed objects can also be machined or easily sanded after printing to smooth the surface [49]. It has been proven that some FDM 3D printed objects have similar mechanical properties as the objects that are manufactured using traditional injection moulding and machining when the same materials are being used. Therefore, FDM 3D printing can provide a cost-effective alternative for producing these parts. Some high-performance thermoplastics can also provide strength that is similar to metal parts. The advantage of using 3D printer thermoplastics over metal is that the thermoplastics products are much lighter than the metals. Some FDM printers have enhanced features such as having multiple printheads which allow for the printing of several different materials within an object.

In recent years, 3D printing has been able to attract attention for use in medical and pharmaceutical applications. It has been adopted by the medical and pharmaceutical industry as a manufacturing method due to its customisability and the ability to create complex shapes with precision. It has successfully been used to produce oral dosage forms [53]. The trend of using 3D printing to produce medicines only started after the approval of the first 3D printed drug by the U.S. Food and Drug Administration (FDA) in 2015. The first 3D printed drug is called Spritam^®^ (levetiracetam). However, this medicine is manufactured using the Zipdose technology based on a powder bed-liquid 3D printing technology [54]. In a powder based 3D printer, a binder solution is required to bind the loose powder together. The binder solution is sprayed onto the successive layers of powder that is being deposited onto the print bed [47]. The traditional methods for producing medicines have very limited process capability and manufacturing flexibility when compared to 3D printing. By using 3D printing, there is much flexibility in designing the tablets. For instance, tablets can be designed to have interesting and complex shapes which can be useful for the fabrication of paediatric medicines or modified-release systems [54,55]. Furthermore, tablets can be designed to contain several drugs that can have different drug release rates. The research work done by Sadia et al. has been able to produce a novel design of a tablet that could accelerate drug release of 3D printed tablets. The 3D printed tablets are made of polymethacrylate and are designed to have built-in channels within the tablet that could increase the surface area/volume ratio of the tablet and also allow media perforation through the structure [55]. The study carried out by Goyanes et al. has also proven that the FDM 3D printing technology is feasible of fabricating personalised tablets containing drug doses tailored to individual patients or tablets with specific drug-release profiles [56]. Apart from that, some research work has been carried out on the possibility of housing multiple medications within a dosage form. This multi-active solid dosage form is called ‘polypill’. The ‘polypill’ can also be designed with having different release profiles [57]. One way to achieve this is by changing the exposed surface area or the geometry of the dosage form and by using more than one kind of carrier with different release characteristics. The external shape and volume/mass ratio of a 3D printed tablet can affect the release profile and pattern. The percentage of infill that can be set during the 3D printing process is one of the parameters that can also affect the drug release patterns [55,58]. Thus, 3D printing has opened up the possibility of combining multiple medications in a personalised tablet. This is particularly useful for patients that are currently taking multiple medications through many separate tablets. Besides that, there is also a flexibility to tailor a specific drug combination or drug release profile according to the patient’s needs. Therefore, 3D printing is able to fabricate these personalised medicines in a very timely and cost-effective manner.

## 5. Coupling HME with FDM 3D Printing

The thermoplastic materials being used in FDM 3D printers need to be in the form of a filament. However, most filaments currently available in the market are not suitable to be used for pharmaceutical applications. This issue has been a major obstacle for FDM 3D printing being used as a main manufacturing method for pharmaceutical products. Apart from that, most traditional polymer excipients that have been used in pharmaceutics do not have the appropriate thermal and mechanical properties that can be used to made into filaments that are needed for FDM 3D printing [59]. Some of the most commonly pharmaceutical grade polymers that can be made into filaments are Polyvinylpyrrolidone (PVP), Polyvinyl alcohol (PVA), and PLA [60]. These are suitable to be made into filaments due to excellent mechanical and thermal properties, and have been used to mix with drugs to produce filaments that can be used in a FDM 3D printer to produce oral dosage forms [61,62]. Filaments that are being used in FDM 3D printers need to be of good quality in order to produce a good quality 3D printed product. During the feeding of the filament into the print head of an FDM 3D printer, the filament will experience tensile and compressive forces caused by the extruding gear, as well as a large amount of heat for melting. The filaments will need to be able to withstand the mechanical and thermal stresses in the FDM print head in order to achieve a good printing quality [49]. Good quality thermoplastic filaments can be effectively produced using the HME method. With twin-screw extrusion in the HME technology, almost any kind of polymer can be mixed during the HME process in order to produce polymeric filaments for the desired applications. Therefore, there has been increasing research interest in the development of different types of filaments from different materials for specific applications using the HME technology. For instance, pure Eudragit has not been a suitable material to be made into FDM printable filaments due to its brittleness. Sadia et al. have been able to develop Eudragit-based filaments by mixing it with a plasticiser (triethyl acetate) and a filler (tricalcium phosphate) using the HME technique [63]. The mechanical properties such as the flexibility of the Eudragit filament have been further improved and the filaments are suitable to be used in FDM 3D printers for the fabrication of tablets. The 3D printed tablets also possess excellent mechanical strength [63]. Due to the robustness of HME in producing novel polymeric filaments for the use in FDM printers, much research has been carried out to explore the possibility of bridging HME and FDM 3D printing to increase the range of polymers that can be used with FDM machines. This approach is seen to be a very efficient and effective way of producing desired materials for FDM printers. It will also further improve the usability of FDM printers across many industries.

**Figure 9:**
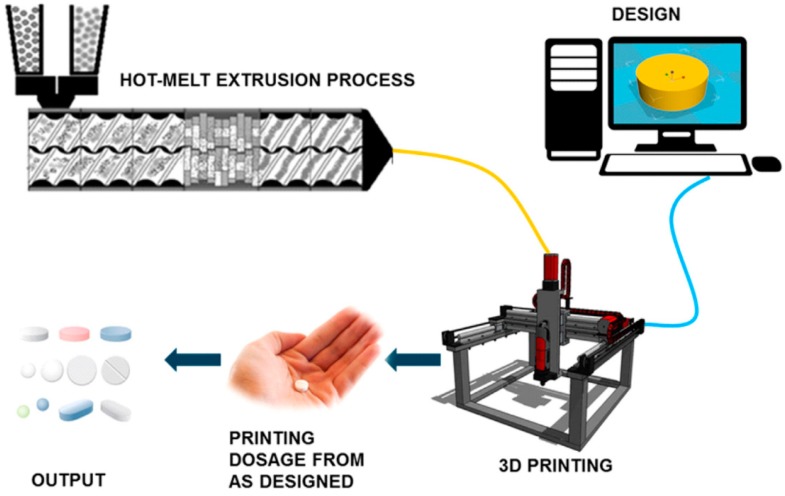
Schematic of a combined Hot-Melt Extrusion (HME) and FDM 3D printing into a single continuous process (Reproduced with permission from reference [16], Elsevier, 2017).

The HME technology and 3D printing technology has shown many advantages when being used in pharmaceutical applications. These two technologies can streamline the complex processes of conventional manufacturing methods for pharmaceutical products. Therefore, there has been some research on combining these two technologies into a single continuous process in order to achieve a more effective and efficient manufacturing process. When these two technologies are coupled into one single process, it opens up the possibility of creating any dosage forms in-house for immediate consumption. This is particularly useful for hospitals in remote areas. Combining these two processes makes the fabrication of dosage forms more cost-effective, efficient and economical. Figure 9 shows a schematic of the HME combined with FDM 3D printing into a single continuous process.

### 5.1. Controlled-Release Dosage Forms

Zhang et al. have been able combine the HME and FDM 3D printing in a single process to produce controlled-release tablets [16]. Different types of pharmaceutical polymers were made into filaments using HME and they were loaded with Acetaminophen as a model drug. The drug release profiles of the 3D printed tablets and the directly compressed tablets were being compared. The study shows that the 3D printed tablets have better extended drug release rates than the directly compressed tablets as they have smoother surfaces and tighter structures. The study has proven that it is possible to produce different formulations required using a single process [16]. Zhang et al. have been able to develop controlled-release 3D printed tablets by using hydroxypropyl methylcellulose (HPMC) as a hydrophilic matrix polymeric material in the drug [64]. HME was used to prepare solid dispersion filaments with an API dispersed in HPMC based matrix. The filaments produced are fed into an FDM 3D printer to produce controlled-release tablets with different structural design in order to obtain different drug release profiles. Nine tablets with the same overall tablet dimensions (diameter, 10 mm; thickness, 4.5 mm) but with different outer shell thicknesses and core densities were 3D printed. The geometry of 3D printed tablets can affect the dissolution and drug release rates. The study has proven that 3D structure design is effective and efficient for optimising controlled drug release rates [64]. Goyanes et al. have also fabricated controlled-release budesonide tablets using FDM 3D printing. The work is to explore the feasibility of using FDM with HME and fluid bed coating to fabricate modified-release budesonide dosage forms. Budesonide is loaded into PVA filaments using HME. An FDM 3D printer is then used to produce capsule-shaped tablets (caplets) containing 9 mg of budesonide. The caplets were then overcoated with a layer of enteric polymer. The 3D printed caplet formulation started to release drug in the mid-small intestine, but release then continued in a sustained manner throughout the distal intestine and colon. This work has demonstrated the possibility of combining FDM 3D printing, HME and film coating for the fabrication of modified-release oral dosage forms [65].

The work by Okwuosa et al. coordinates FDM 3D printing and liquid dispensing for the fabrication of individualised dosage form on demand in a fully automated fashion. This work has the potential of modifying drug dose and release. Polymethacrylate shells (Eudragit EPO and RL) were made using FDM 3D printing and the shells were simultaneously filled using a computer-controlled liquid dispenser loaded with a model drug solution (theophylline) or suspension (dipyridamole). The shell thickness of 1.6mm, and concentric architecture allowed for successful containment of the liquid core whilst maintaining the release properties of the 3D printed liquid capsule. Modifying the shell thickness of Eudragit RL capsule allowed for a controlled extended drug release without the need of formulation change. This type of hard shell capsule is better for taste and odour masking, improving the patient’s compliance. This low cost and versatile approach can be adapted to a wide spectrum of liquid formulation such as small and large molecule solutions. This method also eliminates the need for material compatibility with the high temperature of FDM. This is the first time that an example of a fully automated additive manufacturing process for a liquid capsule with the ability to control the dispense dose has been demonstrated. A dual head FDM 3D printer is used here. However, one of the printing heads has been modified to include a syringe-based liquid dispenser. The drug-free Eudragit filaments were produced using HME. Dimethylaminoethyl methacrylate, butyl methacrylate, and methyl methacrylate (Eudragit EPO) is used for the shell of immediate release dosage forms and Eudragit RL is used for time controlled drug release. The dosage can be controlled by manipulating the dispensed volume and the capsule shell thickness. This work has again proved the capability of FDM producing individualised dosage forms [66].

### 5.2. Sustained-Release Dosage Forms

Chai et al. have used HME to produce hydroxypropyl cellulose (HPC) filaments that are loaded with Domperidone (DOM) to be used in FDM 3D printers. The DOM loaded HPC filament is used to 3D print intragastric floating sustained-release tablets that have a hollow structure, which makes the tablet have a lower density and allows it to float. The hollow structure of the tablet can be achieved by adjusting the shell numbers and the infill percentage of the FDM 3D printing process. The study shows that the capability of FDM 3D printers, a cost-effective platform, to produce hollow tablets is a promising manufacturing method to fabricate intragastric floating drug delivery devices [67]. Okwuosa et al. have also used FDM 3D printers to produce delayed release shell-core gastric resistant tablets. A dual extruder FDM 3D printer has been used to produce an enteric-coated tablet in a single fabrication process. In this study, PVP has been used as a polymer for the core and methacrylic acid polymer for the shell for gastric protection. Similarly, the drug loaded filament for the core and the filament for the shell were manufactured using HME. This work has proven that the FDM 3D printer is able to perform enteric coating to produce delayed release solid dosage forms [61]. Goyanes et al. have been able to use FDM 3D printing to produce modified-release paracetamol tablets without the need of an enteric coating [68]. Again, HME has been used to produce paracetamol-loaded filaments for the use in FDM 3D printers. In this study, paracetamol is mixed with three different grades of excipients hypromellose acetate succinate (HPMCAS) (grades LG, MG and HG) respectively. The HPMCAS-based tablets exhibit delayed release properties, and tablets made from lower pH-threshold release drug faster (LG has the lowest pH threshold of 5.5, MG at 6.0, and HG at 6.5). This study shows that FDM 3D printing is able to produce delayed release tablets without the need for an outer enteric coating. The work by Li et al. has been able to combine HME and FDM to produce a novel controlled-release drug delivery device for diabetic treatment [69]. HME was used to load Glipizide onto PVA to form the drug-loaded filaments. A dual extruder FDM 3D printer was used to produce a double chamber device, where a tablet is embedded within a larger tablet (DuoTablet), and both chambers contains different doses of glipizide. The different concentration of the drug at two chambers is able to achieve a sustained-release profile of the drug. The drug in the external layer is first released and the drug in the second layer will only be released when the first layer has been completely dissolved, delaying the drug release profile of the solid dosage form. This study shows the feasibility of producing a DuoTablet with controlled-release profile in a single process [69].

Verstraete et al. conducted a study to develop high drug loaded (>30%, *w/w*), thermoplastic polyurethane (TPU)-based dosage forms using FDM 3D printing. This is because TPU shown potentials for the manufacturing of highly dosed oral sustained-release matrices via HME [70]. The drug loaded TPU filaments were prepared using HME. Different grades of TPU were mixed with model drugs of different particle size and solubility. Only the TPU-based filaments that exhibit consistent filament diameter, smooth surface morphology and good mechanical properties were used in FDM printers to be printed into tablets. It is proven that TPU-based filaments could be loaded with 60% (*w/w*) fine drug powder. The hot melt extruded hard TPU filaments were successful in producing 3D printed tablets with high concentration of crystalline drug (up to 60%, *w/w*). The drug release profile of the 3D printed tablet were dependent on the matrix composition and the tablet infill degree. The dosage and release rate can be adjusted according to the patient’s needs by change the matrix composition and the percentage of tablet infill. The study shows that TPU-based filaments can offer much formulation freedom for the development of personalised dosage forms [71].

### 5.3. Rapid/Immediate–Release Dosage Forms

Although there is much work that has proven the ability of FDM 3D to produce various drug delivery systems, it has not been a favoured technique when it comes to the fabrication of immediate release tablets. This is because FDM 3D printed tablets generally have a slower dissolution rate when compared to tablets produced using the conventional methods [72]. For instance, the FDM 3D printed Theophylline tablets by Okwuosa et al., which is made from Eudragit^®^ E and PVP with plasticisers required 30 min for the drug to be completely released [62]. However, Kempin et al. explored the possibility of improving the dissolution speed of FDM 3D printed tablets by using different types of polymeric carriers [72]. The study carried out shows that when suitable pharmaceutical grade polymer is being used, FDM can be used to produce immediate release tablets that contains thermolabile drugs. The drug release profiles of pantoprazole sodium tablets made from five different polymers (PVP K12, PEG 6000, Kollidon^®^ VA64, PEG 20000 and poloxamer 407) were compared. The drug was loaded onto each polymer using the HME technology to produce drug-loaded filaments. The results show that the drug incorporated in PVP has the fastest release and can be completed within 10 min. The dissolution rate can also be further improved by reducing the infill rate during printing to increase the porosity of the tablet [72]. Apart from that, the study has also proven that FDM 3D can produce tablets at processing temperatures below 100 °C. FDM 3D printers are known to have very high printing temperatures (170–230 °C) due to the high melting temperature of the traditional polymeric filaments used and could be inappropriate for the incorporation of thermos-sensitive drugs. However, the processing temperature of FDM 3D printers can be lowered when suitable polymers can be made into filaments chosen as a drug carrier. Kollamaram et al. have also been able to produce immediate release formulations (Ramipril) through FDM 3D printing at a lower printing temperature. The drug is mixed with Kollidon VA64 and Kollidon 12PF to achieve the immediate release effect. Drug loaded filaments were first produced using the HME technology at 70 °C. The 3D printer was able to print the tablets from the filaments produced at 90 °C and there was no signs of thermal drug degradation on the printed tablets. The work has proven that FDM 3D printing can also be used to produce thermolabile and low-melting point drugs when the drug is mixed with appropriate excipients [73]. The study by Solanki et al. have produced FDM 3D printed tablet for rapid drug release. The model drug used is haloperidol and the drug loaded filaments were prepared using HME. Different type of polymers were used to be made into filaments (Kollidon^®^ VA64, Kollicoat^®^ IR, Affinsiol TM 15 cP and HPMCAS). Tablets of 100% and 60% infill were printed at 210 °C and dissolution test were conducted at pH 2 and 6.8. For the 60% infill tablet, complete drug release was achieved in 45 min, which was significantly faster than the 100% infill tablet. This is because the 60% infill tablet is porous and the 100% tablet is non-poroous. The filaments made of Kollidon^®^ VA-64-Affinisol TM 15 cP mixtures were flexible and had optimum mechanical strength for 3D printing. The 1:1 mixture of Kollidon^®^ VA-64 and Affinisol TM 15 cP was the most suitable polymer system to be used for 3D printing and rapid drug release. The study has proven that FDM 3D printing can also be used to fabricate immediate release dosage forms [74].

### 5.4. Drug Loaded Implants

Kempin et al. have used HME to produce quinine loaded filaments that can be used in FDM 3D printers to produce drug loaded implants. The drug release of different type of polymers (Eudragit^®^ RS, Polycaprolactone (PCL), poly(l-lactide) (PLLA), and ethyl cellulose (EC)) were being compared. The study shows that PCL has the fastest drug release whereas Eudragit and EC have the slowest drug release. The study has proven the feasibility of FDM 3D printing to manufacture drug loaded implants as FDM 3D printing can produce a product of almost any shape, which could be designed to function as an implant, and at the same time, be able to incorporate a desired drug delivery system [75]. Besides that, Genina et al. used ethylene vinyl acetate (EVA) as a drug carrier to produce drug loaded EVA filaments using HME. The EVA filaments were loaded with indomethacin using HME. The filaments produced were used in FDM 3D printers to fabricate custom-made T-shaped intrauterine system (IUS) and subcutaneous rods (SR). The aim of this work is to investigate the printability of different grades of EVA copolymers as new feedstock material for FDM 3D printing in the fabrication of drug delivery devices. The IUS and SR were successfully printed at a temperature just above the melting point of the drug. The study has proven that EVA 5 polymer has the required properties to be made into filaments for the FDM 3D printing of drug delivery systems such as IUS and SR devices [76]. Holländer et al. have also been able to produce indomethacin loaded PCL filaments using HME. The drug loaded PCL filaments were then used in FDM 3D printers to produce drug-containing T-shaped prototypes of IUS. This study shows that the fabrication of controlled-release implantable devices can be achieved using FDM 3D printing and HME technology [77]. Fu et al. have been able to use FDM 3D printing to produce personalised progesterone-loaded vaginal rings. Progesterone was mixed with Polyethylene Glycol (PEG) 4000 to form a solid dispersion and was cut into pieces. The mixture of PLA and PCL (8:2), Tween 80 and the progesterone-loaded PEG were mixed in a desktop single screw extruder and hot melt extruded in the form a filaments. The filaments were fed into FDM 3D printers to print vaginal rings in the shape of “O”, “Y”, or “M”. The work shows that “O” ring had a higher dissolution rate due to its higher surface area/volume ratio. The vaginal rings also have long-term sustained-release of progesterone for more than 7 days with diffusion-controlled release behavior. It is again been proven that HME and FDM 3D printing is an effective method to prepare personalised and customised medications [78].

### 5.5. Dosage Forms with Multiple Medications

**Figure 10:**
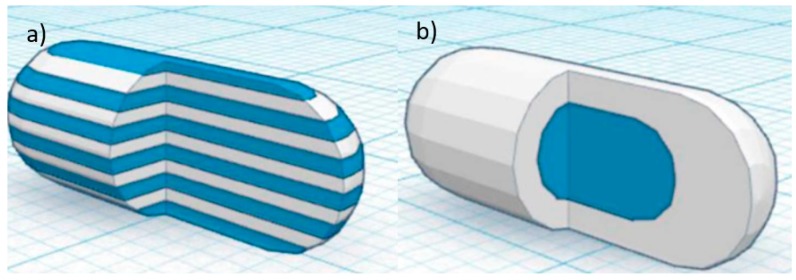
A 3D computer-aided design (CAD) model of capsule-shaped oral drug delivery device (**a**) multilayer device (**b**) two-compartment caplet (Duocaplet) (Reproduced with permission from reference [83], American Chemical Society (ACS), 2015).

In recent years, there has also been increasing interest in developing a dosage form that contains multiple medications [57,79,80]. Having more than one API in a dosage form can improve patient compliance and cost-effectiveness [81]. This type of multi-active solid dosage form, also known as polypill, is particularly useful for patients with complicated diseases, who are required to take multiple medications in a day. The polypill can reduce the frequency of medications intake in a day for the patient. The work by Gioumouxouzis et al. discovered the capability of FDM 3D printing to fabricate dosage form containing two APIs. The bilayer dosage form contains two anti-diabetic drugs (metformin and glimepride). Metformin was mixed with Eudragit^®^ RL to achieve a sustained-release effect and glimepride was mixed with polyvinyl alcohol (PVA) for an immediate release system. In this study, the two APIs were incorporated into two different polymeric carriers to achieve distinct release characteristics. The 3D printed bilayer dosage form allows for the simultaneous usage of two APIs that are administered at different times of the day according to the patient’s needs. The drug-loaded filaments were also manufactured using the HME technology. This study shows that FDM 3D printing can be a promising technique for the fabrication of complex personalised medicine consisting of different APIs that exhibit different release patterns [82]. Goyanes et al. used a dual-extruder FDM 3D printer to fabricate two different capsule-shaped oral drug delivery devices. One is a multilayer device and the other is a two-compartment caplet (Figure 10). The multilayer device is designed in such a way, that two adjacent layers contain different type of drugs. The two-compartment caplet is a smaller caplet embedded in a larger caplet (Duocaplet), with each compartment containing a different drug. In this study, PVA-based filaments were used and they were loaded with paracetamol or caffeine. The filaments were prepared using the HME method. The drug release study shows that for the multilayer device, drug release rates were similar for both drugs. However, if one layer has a higher drug loading than the other, the release rate will be faster for higher drug loading. For the Duocaplet, the drug on the external layer is being released first. The time lag of the release of drug in the inner caplet depends on the characteristics of the external layer. The study has proven that tablets that are 3D printed in different shapes can have different release profiles [83].

**Figure 11:**
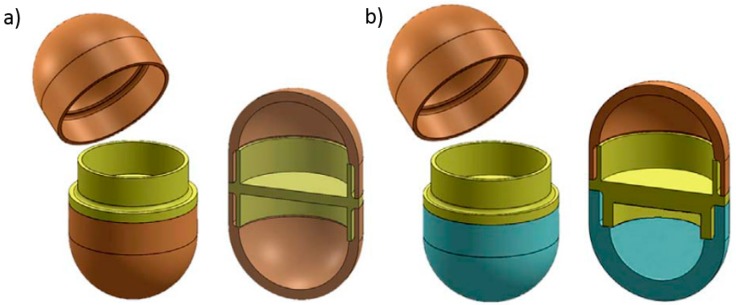
Two-compartment capsular device (**a**) compartments with the same wall thickness (**b**) compartments with different wall thickness (Reproduced with permission from reference [84], Elsevier, 2017).

Maroni et al. used FDM 3D printing to fabricate a two-compartment capsular device for two-pulse oral drug delivery purposes. The study carried out in this work involves the combination of two compartments with different wall thickness and compositions. Promptly soluble, gastroresistant and swellable/erodible compartments were developed, which allowed for immediate, enteric and pulsatile release to be achieved respectively. Therefore, different types of polymeric filaments have been used to achieve different effects. The polymeric filaments were prepared using twin-screw extrusion. Apart from different materials, the compartments were also made to be of different wall thickness (600 µm and 1200 µm) (Figure 11). Such a device is intended to yield multiple release kinetics. The two separate compartments are to be filled either with different APIs or with varied doses and/or formulations of one particular drug. These devices with different compartment characteristics show two pulse release patterns. These two-compartment capsular devices are able to convey incompatible drugs or different drug formulations [84].

Tagami et al. were able to fabricate 3D-printed composite tablets consisting of two components, a drug and a filler, using a dual head FDM 3D printer. PVA containing calcein was used as the drug component and drug-free PVA or PLA was used as the water-soluble or water-insoluble filler respectively. The filaments were produced using a twin-screw extruder. Different designs of the tablets were being used. There were eight kinds of calcein-loaded PVA/PVA composite tablets and four kinds of calcein-loaded PVA/PLA tablets that were 3D printed. All composite tablets were designed to have different surface areas of calcein-PVA but all have a drug component/filler component ratio of 2/3 (*v/v*). The composite tablets showed various drug release profiles. The higher surface area of calcein-PVA had a higher dissolution rate. The study shows that the polymer filler component was effective in controlling drug release and will be particularly useful for personalised medicine [85]. The study carried out by Melocchi et al. has successfully proven the feasibility of FDM 3D printing in the manufacturing of capsular devices for oral pulsatile release using HPC-based filaments. Pharmaceutical grade HPC-based filaments are not commercially available and they were produced using HME. The capsular device produced using FDM 3D printing exhibited satisfactory physico-technological properties. The release test of the capsular device shows that there is a lag phase before rapid and quantitative liberation of the drug. The morphological of the device when in contact with water and the release performance are comparable to the analogous systems fabricated using injection moulding. Since it is possible to rapidly prototype capsular devices using FDM 3D printing, the screening of formulation and design characteristics of these devices are also expected to be accelerated [86].

### 5.6. Complex-Shaped Medicines

As FDM 3D printing can be used to produce complex personalised medicines, Arafat et al. have been able to use 3D printers for flexible on-demand precision tailored dose adjustment [87]. This type of medication has been beneficial for anticoagulant therapy. For anticoagulation therapy, regular coagulation monitoring and dose modification is required in order to ensure the patient’s International Normalised Ratio (INR) remains in the target range. Therefore, dose modification is required from time to time, depending on the patient’s condition, as the patient’s INR profile may be constantly changing. The 3D printing technology is able to produce a precise dose and has been a platform to fabricate personalised medicine with the required dose. The technology allows the administration of the lowest effective dose of the drug to maintain the target INR due to its flexible and precise manufacturing capability. Arafat et al. have used FDM 3D printers to produce a tailored dose of a narrow therapeutic index drug, warfarin, and have successfully fabricated this purposefully designed solid dosage form in ovoid tablets. The study showed that FDM 3D printing can be used to engineer and control the dose of immediate release warfarin tablets by controlling the mass and volume of the 3D printed tablet. The drug loaded filaments were produced using a twin-screw hot-melt extruder and Triethyl citrate (TEC) was mixed into the drug loaded filament formulation in order to lower to the glass transition temperature of the filament [87].

Apart from that, the work done by Scoutaris et al. has also proven that a continuous process of HME coupled with FDM 3D printing can be used to prepare chewable tablets in the shapes of “Starmix^®^” sweets [54]. This is a very effective method to produce paediatric medicines as the HME is able to provide taste masking of bitter APIs, and FDM 3D printing has the capability to produce complex shapes tablets which could attract the attention of children. In this study, the drug used is Indomethacin (IND) and the thermoplastic polymer used is HMPCAS. HME has efficiently dispersed the IND in the HPMCAS matrix, providing excellent taste masking efficiency. FDM 3D printing has shown to have high reproducibility, accuracy and content uniformity of the Starmix^®^ shapes. This study has proven an alternative route of producing palatable paediatric dosage forms. Jamróz et al. carried out a study that used FDM 3D printer to prepare orodispersible films (ODFs) containing a poorly water soluble substance, Aripiprazole. The study proves the feasibility of fabricating ODFs using FDM technology and to improve the dissolution rate of the poorly water-soluble substance through 3D printed films. The Aripiprazole loaded PVA filaments were prepared using HME. The study shows that amorphisation of the aripiprazole and the porous structure of 3D printed film is able to improve its dissolution rate. The mechanical properties of the 3D printed films were also comparable to the films prepares using the casting method [88].

Beck et al. also carried out a study regarding an innovative approach to produce customised drug delivery systems using FDM 3D printing. This work produced a multi-functional drug delivery system, which is a solid dosage form loaded with nano-sized carriers. The possibility of fabricating solid dosage form containing drug-loaded nanocapsules using FDM 3D printing has been discovered. This work integrates the FDM 3D printing technology and nanotechnology to produce innovative nanomedicines. PCL and Eudragit^®^ RL100 (ERL) filaments were produced using HME. The 3D printed tablets were then loaded with polymeric nanocapsules. The study proposed a new platform for the development of oral dosage forms, biodegradable implants with tailored dose and drug release profiles, as personalised medicines [89].

## 6. Conclusions

HME is gradually becoming a preferred manufacturing technology in the pharmaceutical industry due to its many advantages over conventional fabrication techniques. It has been proven to be a very efficient technology in producing different drug delivery systems such as the solid dispersion, modified drug release systems, and targeted drug release systems. One major advantage of the HME is that it is more environmentally friendly as it is a solvent-free process. It is a very effective improving the bioavailability and solubility of poorly soluble drugs, as well as taste masking the bitterness of APIs. FDM 3D printing has also been proven to be a versatile 3D printing method. It is a very cost-effective 3D printing method and have been used across various disciplines. FDM 3D printing has a great potential to be the main manufacturing method for pharmaceutical devices, in particular personalised medicines and implants, due to its flexible manufacturing capability which allows for the fabrication of pharmaceutical products with nearly any shape. When being used in pharmaceutics, FDM has the capability to produce complex shape dosage forms, which could play a role in the release pattern of a drug. The advantages of using FDM in the production of pharmaceutics include design flexibility, cost-effectiveness and high reproducibility. As FDM takes thermoplastic filaments as printing materials, the filaments can be produced using HME. Drug loaded filaments can be extruded from the HME and then fed directly to the FDM 3D printer. Therefore, the HME technology and FDM can be combined into a single continuous process for higher efficiency. The combined process provides an automatic production process and could limit material loss. When these two technologies are coupled into one single process, it opens up the possibility of creating any dosage forms in-house for immediate consumption. This is particularly useful for hospitals in remote areas. This continuous process also offers the flexibility in producing formulations based on the personal needs of patients. The personalised medicines can be produced in house for immediate use. Combining these two processes makes the fabrication of dosage forms more cost-effective, efficient and economical. The HME and FDM combined process has opened up the possibility of producing any types of formulations and drug delivery systems and is a novel technique to be used in the production of pharmaceutical dosage form and medical devices.

## Figures and Tables

**Table 1:** Advantages and disadvantages of single-screw and twin-screw extruder.

Type of Extrusion	Advantages	Disadvantages
Single-screw extrusion	Mechanical simplicity Low maintenance Low cost	High tendency of overheating Not suitable for heat-sensitive materials Poor mixing
Twin-screw extrusion	Easier material feeding Higher kneading and dispersing capacity Lower tendency to overheat Higher process productivity and flexibility Better control of process parameters Enhanced mixing	High input energy Not suitable for shear-sensitive materials

**Table 2:** Printing temperature, properties and applications of different thermoplastic materials used in FDM 3D printing.

Thermoplastic Materials	Acrylonitrile Butadiene Styrene (ABS)	Polylactic Acid (PLA)	Polyether Ether Ketone (PEEK)	Polyether Imide (PEI)
Printing temperature (°C)	220–250	190–220	350–400	355–390
Properties	High strength, flexibility and durability	Biodegradable, biocompatible, brittle	High mechanical strength, toughness, flexibility, chemical and radiation resistance, self-extinguishing properties	High specific strength (strength-to-weight ratio), flame resistance, chemical resistance
Applications	Pipe systems, automotive, toys, electronic casing	Biomedical, drug delivery systems, food packaging	Electronics, aerospace, automotive, mechanical and medical parts	Medical, chemical instruments, aerospace, automotive, electronics
